# The bHLH-PAS gene *hlh-34* is expressed in the AVH, not AVJ interneurons

**DOI:** 10.17912/micropub.biology.000467

**Published:** 2021-09-28

**Authors:** Steven J. Cook, Berta Vidal, Oliver Hobert

**Affiliations:** 1 Columbia University, Department of Biological Sciences, HHMI, New York, NY

## Abstract

Single neuron-specific drivers are important tools for visualizing neuron anatomy, manipulating neuron activity and gene rescue experiments. We report here that genomic regions upstream of the *C. elegans* bHLH-PAS gene *hlh-34 *can be used to drive gene expression exclusively in the AVH interneuron pair and not, as previously reported, the AVJ interneuron pair.

**Figure 1. AVH and AVJ neurons and hlh-34 expression:**
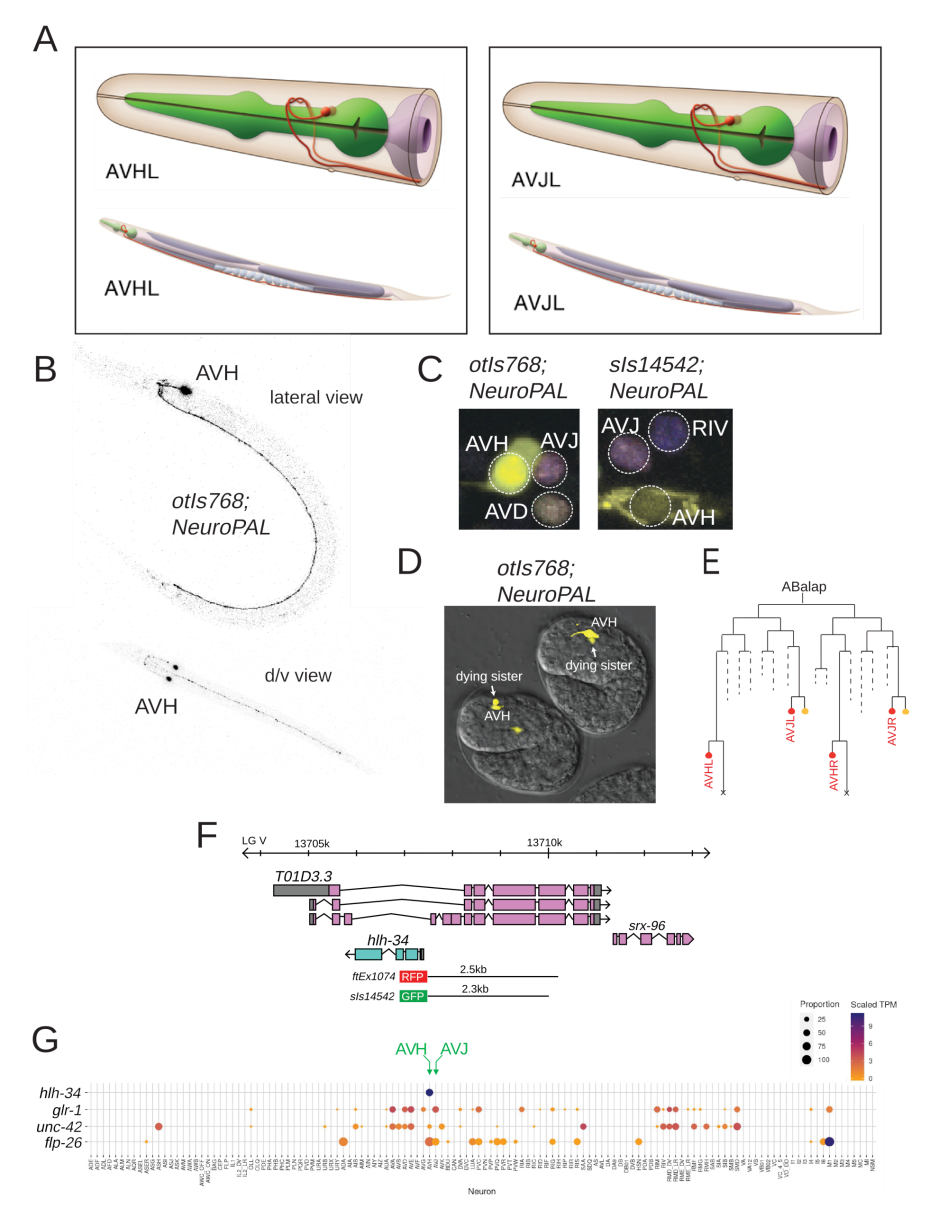
**A:** AVH and AVJ display a nearly identical position and axon trajectory in the lateral ganglion of *C. elegans.* Images used with permission from Wormatlas. **B:** Whole-animal lateral and dorsoventral overviews of *hlh-34prom::gfp* (*otIs768* transgene) expressing young larvae. Animals imaged in a NeuroPAL (*otIs669)* background with only GFP emission shown for simplicity. **C:** Expression pattern of *otIs768* and *sIs14542* transgenic reporters in a NeuroPAL (*otIs669)* background. GFP signal is only observed in the AVH neuron. **D:** Embryonic expression pattern of *otIs768*. At ~360 minutes the GFP signal is observed in the AVH neuron and its dying sibling cell. All animals were imaged in aNeuroPAL (*otIs669)* background with only GFP emission shown for simplicity. **E:** Lineages of AVH/J in red. CEPsoDL/R shown in orange. Dying siblings of AVH shown as ‘x’. **F**: *hlh-34* locus and promoter fragment used in the *sIs14542* transgene. Coordinates are V:13709947..13712813 in WS140 and V:13707915..13707382 in WS280. The size of the promoter used in the *leEx1692* strain, kindly provided by Ian Hope, could not be unambiguously determined. The strain used in the first publication on *hlh-34* (Cunningham *et al.* 2012) used 2.5kb upstream of *hlh-34*. **G:** Heatmap of scRNA expression (Taylor *et al.* 2021), showing *hlh-34* expression and other markers of AVH and AVJ identity.

## Description

bHLH-PAS transcription factors are a metazoan-specific family of transcription factors with diverse functions within and outside the nervous system (Kewley *et al.* 2004). *Caenorhabditis elegans* contains five bHLH-PAS proteins: (1) *ahr-1*, an ortholog of the aryl hydrocarbon receptor(Powell-Coffman *et al.* 1998)*;* (2) *aha-1,* an ortholog ofthe Aryl Hydrocarbon Receptor Nuclear Translocator (ARNT), which is a common dimerization partner for many, but not all bHLH-PAS genes (Powell-Coffman *et al.* 1998);(3) *hif-1*, an ortholog of hypoxia-inducible factor HIF1alpha (Jiang *et al.* 2001);(4) CKY-1, which orthology prediction tools consider to be an ortholog of NPAS4, even though its bHLH domain is very degenerate (nevertheless, CKY-1 heterodimerizes, like other *C. elegans* bHLH-PAS proteins, with the common AHA-1 partner protein in yeast 2 hybrid assays (Grove *et al.* 2009)); (5) *hlh-34,* which Marvvel (Wang *et al.* 2019) and Ortholist (Shaye and Greenwald 2011) predict to be an ortholog of both the NPAS1/3 and the SIM1/2 subgroups of bHLH-PAS proteins (Yan *et al.* 2014).However,HLH-34 contains neither of the domains that are found, in addition to the canonical bHLH and PAS domains, in either vertebrate NPAS (PAS_11 domain) or SIM (SIM_C) proteins. HLH-34 may therefore reflect an ancestral version of both subgroups.

In this paper, we re-assess the previously reported expression pattern of the *hlh-34* gene. An intriguing previous study that analyzed feeding control in *C. elegans* used 2.5kb of the *hlh-34* promoter in a reporter gene construct to assess expression of the gene(Cunningham *et al.* 2012). This reporter was described to be expressed exclusively in a single neuron pair that the authors tentatively identified as the AVJ neurons (Cunningham *et al.* 2012). Based on rescue experiments that the authors performed with the *hlh-34* promoter, feeding behavior functions were ascribed to AVJ (Cunningham *et al.* 2012). Moreover, again based on the expression assignment of the *hlh-34* promoter, an interesting recent study implicated the AVJ neurons in cold tolerance behavior (Takagaki *et al.* 2020). Lastly, the neurogenin homolog *ngn-1* was recently reported to function in the AVJ neurons, based on its regulation of *hlh-34* reporter expression (Christensen *et al.* 2020).

However, AVJ and its neighboring AVH neuron pair are two bilaterally symmetric neuron pairs that are notoriously difficult to distinguish based on their almost identical cell body position and neurite trajectory (White *et al.* 1986)(**Fig.1A**).

In the context of studying fate specification of the AVJ and AVH neurons (Berghoff *et al.* 2021), we obtained an *hlh-34* promoter-based reporter line from Ian Hope, *leEx1692,* established in initial attempts to broadly studying gene expression (Hope *et al.* 1996). We found that this transgene is expressed in a single head neuron pair, extending its neurite along the ventral nerve cord (**Fig.1B**), consistent with this neuron being either the AVJ or AVH neuron pair. After chromosomal integration, we crossed this transgene (*otIs768)* with NeuroPAL, a transgene that provides a multicolor bar code for all neurons in the *C. elegans* nervous system, with distinct color codes for AVH and AVJ (Yemini *et al.* 2021). We find that the *hlh-34prom::gfp* signal from the *otIs768* transgene overlaps with the AVH and not the AVJ signal(**Fig.1C**). We validated this assessment by crossing *otIs768* with an AVH-expressed marker that is not in AVJ (*unc-42::rfp*) and an AVJ expressed marker, that is not in AVH (*glr-1prom::rfp*). We observed an overlap with *unc-42:rfp* but not *glr-1prom::rfp*. We also obtained another *hlh-34* promoter *gfp* reporter from the Moerman & Baillie groups, *sIs14542* (Hunt-Newbury *et al.* 2007) (**Fig.1F**).Using NeuroPAL, we confirmed *sIs14542* expression in AVH, but not AVJ (**Fig.1C**).

We further corroborated *hlh-34prom* expression in AVH by observing that in the embryo, expression of *otIs768* is first transiently observed in 4 cells, two of which show signs of cell death (**Fig.1D**); later in embryogenesis, and then during all stages of postembryonic and adult development, expression becomes restricted to 2 cells. This is consistent with expression in the bilateral AVH neuron pair, since their two sisters cells are, unlike the sisters of the AVJ neuron pair, destined to die by apotosis (Sulston *et al.* 1983) (**Fig.1E**).

Lastly, single cell RNA profiling of the entire *C. elegans* nervous system reveals strong and selective expression of *hlh-34* transcripts exclusively in the AVH neuron (Taylor *et al.* 2021)(**Fig.1G**). The scRNA data not only confirm *hlh-34* expression in AVH but also corroborates the expression of the markers that we used to distinguish AVH from AVJ (**Fig.1G).** Since all previously described *hlh-34* reporter constructs only encapsulate parts of the 5’ region of the gene and because the gene has an unusual location in the intron of another gene (**Fig.1F**), the scRNA data represent an important, independent validation of the assignment of the existing reporter gene patterns to AVH and not AVJ.

The expression of the *hlh-34* promoter in AVH rather than AVJ indicates that functions previously ascribed to the AVJ neuron need to be re-ascribed to the AVH neuron. This applies to the above-mentioned feeding behavior function initially ascribed to AVJ (Cunningham *et al.* 2012) which now has to be assigned to AVH. Similarly, the implication of AVJ in cold tolerance behavior, based on the expression overlap of a gene involved in cold tolerance, *xdh-1*, and *hlh-34prom::rfp,* and based on the rescue of the *xdh-1* mutant phenotype with an *hlh-34prom* driver (Takagaki *et al.* 2020), needs to be re-assigned to the AVH neuron. Lastly, the function of the *ngn-1* bHLH gene of regulating *hlh-34* (Christensen *et al.* 2020) needs to be re-assigned to AVH.

In conclusion, the 5’ region of the *hlh-34* gene is a useful tool to gain genetic access to the AVH neuron.

## Methods

Strains were examined with a laser scanning LSM 880 microscope.

## Reagents

The following transgenes were used:

OH16483: *unc-119(ed3); otIs768[hlh-34prom::gfp, unc-119(+)]*

BC15839: *dpy-5(e907); sIs14542[hlh-34prom::gfp, dpy-5(+)]*

OH15363: *otIs669[NeuroPAL]; him-5(e1490)*
